# Temperature-Dependent Modulation of Chromosome Segregation in *msh4* Mutants of Budding Yeast

**DOI:** 10.1371/journal.pone.0007284

**Published:** 2009-10-09

**Authors:** Andrew Chi-Ho Chan, Rhona H. Borts, Eva Hoffmann

**Affiliations:** 1 MRC Genome Damage and Stability Centre, University of Sussex, Falmer, United Kingdom; 2 Department of Genetics, University of Leicester, Leicester, United Kingdom; National Cancer Institute, United States of America

## Abstract

**Background:**

In many organisms, homologous chromosomes rely upon recombination-mediated linkages, termed crossovers, to promote their accurate segregation at meiosis I. In budding yeast, the evolutionarily conserved mismatch-repair paralogues, Msh4 and Msh5, promote crossover formation in conjunction with several other proteins, collectively termed the Synapsis Initiation Complex (SIC) proteins or ‘ZMM’s (Zip1-Zip2-Zip3-Zip4-Spo16, Msh4-Msh5, Mer3). *zmm* mutants show decreased levels of crossovers and increased chromosome missegregation, which is thought to cause decreased spore viability.

**Principal Findings:**

In contrast to other ZMM mutants, *msh4* and *msh5* mutants show improved spore viability and chromosome segregation in response to elevated temperature (23°C versus 33°C). Crossover frequencies in the population of viable spores in *msh4* and *msh5* mutants are similar at both temperatures, suggesting that temperature-mediated chromosome segregation does not occur by increasing crossover frequencies. Furthermore, meiotic progression defects at elevated temperature do not select for a subpopulation of cells with improved segregation. Instead, another ZMM protein, Zip1, is important for the temperature-dependent improvement in spore viability.

**Conclusions:**

Our data demonstrate interactions between genetic (*zmm* status) and environmental factors in determining chromosome segregation.

## Introduction

During gamete production, germline cells undergo a specialized cell division (meiosis) where two consecutive nuclear divisions follow a single DNA replication event thereby reducing the chromosome number by half. To ensure that each gamete inherits an entire complement of chromosomes, organisms employ an array of different mechanisms, all of which rely on partner recognition followed by their separation (‘segregation’) at the first meiotic division.

Many, but not all, organisms depend upon the formation of chiasmata between the segregating partners, termed homologous chromosomes (homologs) [1]. Chiasmata have been proposed to counteract the pulling forces that are generated when kinetochores attach to microtubules emanating from opposite spindle poles, thereby facilitating the inactivation of the spindle assembly checkpoint once homologs are bioriented [2], [3].

In budding yeast, the precursors to chiasmata, crossovers, are promoted by the Synapsis Initiation Complex (SIC) proteins, also termed the ZMM ensemble (Zip3/Zip1/Zip2-Zip4-Spo16, Msh4-Msh5, and Mer3). Crossovers are generated from a subset of meiotic recombination events and are preceeded by specific double-strand break repair intermediates, including single-end invasions [4] and joint molecules/double Holliday Junctions [5], [6], [7].

Holliday Junctions are substrates of the mismatch-repair paralogues, Msh4 and Msh5 [8]. Msh4 and Msh5 function as a heterodimer during meiosis [9] and do not contain the mismatch-repair binding domain found in other MutS paralogues [10], [11]. Consistent with this, the absence of *MSH4* or *MSH5* influences neither mitotic post-replicative mismatch repair nor meiotic heteroduplex DNA repair, since no significant appearance of post-meiotic segregation events are observed [12], [13]. Furthermore, the human MSH4-MSH5 heterodimer does not show a preference for mismatch-containing heteroduplex DNA over homoduplex DNA [8].

Msh4-Msh5 may promote crossing over by protecting crossover-specific intermediates from being resolved as noncrossovers, for example by Sgs1/BLM [14], [15]. In turn, formation of Msh4-Msh5 foci on meiotic chromosomes depends upon synaptonemal complex proteins, including Zip3 and Zip1.

In the absence of any one of the *zmm* genes, crossover frequencies are reduced, but not abolished [16]. The decreased levels of crossovers are generally thought to cause increased levels of homolog missegregation, resulting in decreased viability of gametes, known as spores in budding yeast. Several observations support the notion that the decreased spore viability is due to increased missegregation of homologous chromosomes. First, when assessed genetically, many of the mutants display increased non-disjunction of homologous chromosomes (reviewed in [17]). Second, partial restoration of crossover levels in *zmm* mutants by mutation of *SGS1/BLM* helicase improves segregation and spore viability [14], [15]. Third, spore viability is decreased when precursors to crossovers, double-strand breaks, are decreased by mutation of Spo11, a topoisomerase II-type protein that catalyzes the DSBs [18]. Fourth, the *zmm* mutants analysed show an increase in the formation of non-exchange chromosomes, particular of smaller chromosomes [19], [20]. This, together with the observation that artificial or sequence-diverged (homeologous) non-exchange chromosome ‘pairs’ in budding yeast missegregate ∼10–20% of meioses (compared to <1% for exchange pairs in wild-type cells) indicate that the generation and subsequent missegregation of non-exchange chromosome pairs contribute to spore death [21], [22], [23], [24].

Here, we have identified temperature as a modulator of chromosome segregation in *msh4* and *msh5* mutant strains. We show that this occurs in different budding yeast strains, without any apparent concomitant increase in crossover levels. We identify Spo11 and Zip1 as important factors for this phenotype.

## Results

### Temperature modulates spore viability in *msh4* and *msh5* mutants

**Figure 1:**
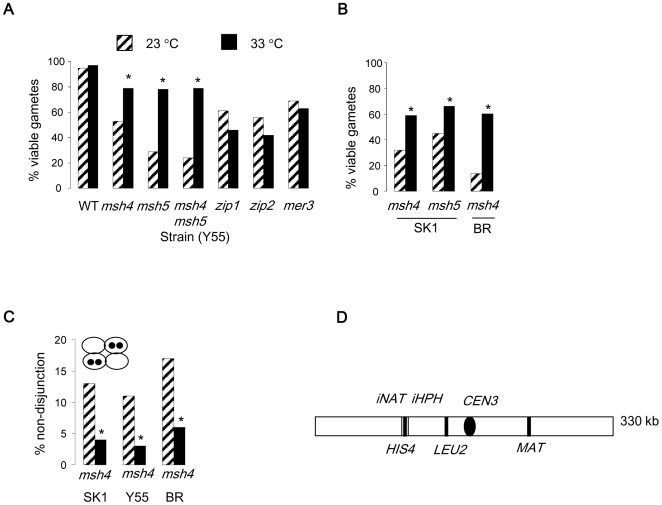
Chromosome segregation and spore viability in the *msh4* and *msh5* mutants is modulated by temperature. (A) Spore viabilities of *zmm* mutants in the Y55 strain background and of *msh4* in BR and SK1 (B). Strains are given in Supplementary Table S1 and viable-spore class distributions in Table 1 (Y55) and Table 2 (SK1). Spore viability was measured by counting viable spores in dissected tetrads. (C) Assessment of meiosis I non-disjunction in spores containing LacO or TetO labelled chromosome IIIs and expressing LacI-GFP or TetR-GFP, respectively. More than 150 tetrads containing four distinct GFP foci were examined using standard fluorescence microscopy. For isogenic wild-type strains, the missegregation frequencies were less than 1/150 (data not shown). The asterisks indicate statistically significantly improved viability frequencies (A and B) and improved segregation (C) compared to 23° (P<0.01, G-test for homogeneity). Hatched bars represent assays at 23° and black bars at 33°.

*zmm* mutants show decreased levels of crossovers at both 23 and 33°C, with a more severe defect at 33°C [16]. Nevertheless, *msh4* and *msh5* mutants showed improved gamete viability at 33°C (Figure 1A and B). This was not the case for the remaining *zmm* mutants (Tables 1 and 2). At 23°C, all of the *zmm* mutant strains showed the characteristic increase in the two- and zero-viable spore classes, consistent with meiosis I non-disjunction (Table 1). The Y55 *msh5* and the *msh4 msh5* mutants displayed a more severe decrease in the proportion of four-viable spored tetrads compared to the *msh4* strain at this lower temperature. Since Msh5 has previously been suggested to be important for chromosome segregation in crossover-defective *mms4 mlh1* strains [25], it is possible that Msh5 has a role independently of Msh4. If so, this is strain-specific as no differences in gamete viability between the *msh4* and *msh5* strains was observed in SK1 (Table 2). Regardless of this, the temperature-dependent improvement in gamete viability was observed in all mutants defective for *MSH4*, *MSH5* or both, irrespective of strain background. Thus, our data suggest two temperature-dependent processes: Msh5 may have a strain-specific chromosome segregation function at 23°C, whereas elevated temperature (33°C) positively impacts chromosome segregation in the absence of Msh4 or Msh5. We focus on the latter phenotype.

**Table 1:** Gamete viability and sporulation frequencies in *zmm* mutants of Y55.

Strain nr.	Genotype	Temp	Category					n	% viability	% sporulation
		(°C)	4∶0	3∶1	2∶2	1∶3	0∶4			
ERY103	Wild type	23	**88**	7	**3**	1	**1**	1987	95	57
		33	**90**	9	**1**	0	**0**	265	97	82
ERY137*	*msh4*	23	**32**	8	**25**	8	**27**	1475	53	45
		33	**63**	11	**15**	3	**8**	421	79	76
		37	**65**	10	**12**	4	**9**	704	80	12
		39	**64**	18	**9**	2	**7**	425	82	3
ERY320*	*msh5*	23	**3**	16	**19**	18	**44**	88	29	58
		33	**60**	18	**8**	1	**13**	78	78	68
ERY432*	*msh4 msh5*	23	**4**	13	**20**	2	**61**	58	24	51
		33	**62**	11	**16**	3	**8**	60	79	59
ERY340*	*zip1*	23	**24**	17	**42**	13	**4**	92	61	48
		33	**6**	18	**44**	19	**13**	90	46	32
ERY254	*zip2*	23	**39**	5	**25**	5	**26**	44	56	78
		33	**27**	2	**25**	2	**44**	48	42	43
ERY319	*mer3*	23	**48**	14	**19**	2	**17**	64	69	44
		33	**37**	24	**13**	6	**20**	62	63	17

* significantly different distributions (P<0.017) of viable spore classes (G-test) and proportion of four-viable spores (t-test) at 33°C compared to 23°C.

**Table 2:** Gamete viability and sporulation frequencies in *zmm* mutants of SK1.

Strain nr.	Genotype	Temp.	Category					n	% viability	% sporulation
		(°C)	4∶0	3∶1	2∶2	1∶3	0∶4			
NKY3220	Wild type	23	**91**	6	**3**	0	**0**	110	97	91
		33	**93**	6	**1**	0	**0**	109	98	89
NKY3227	*msh4* *	23	**12**	9	**23**	8	**48**	104	32	73
		33	**44**	5	**20**	10	**21**	101	59	16
NKY3228	*msh5* *	23	**24**	7	**23**	16	**30**	107	45	68
		33	**49**	6	**21**	10	**14**	103	66	21
NKY3229	*mer3*	23	**23**	4	**32**	6	**35**	100	44	81
		33	**18**	3	**39**	8	**32**	100	41	8
NKY3224	*zip1*	23	**34**	7	**21**	0	**38**	108	50	67
		33	**24**	14	**30**	2	**30**	108	51	14
NKY3225	*zip2*	23	**38**	5	**25**	5	**27**	44	56	76
		33	**27**	2	**25**	2	**44**	48	42	12
NKY3226	*zip3*	23	**22**	5	**33**	18	**22**	110	47	85
		33	**19**	7	**35**	10	**29**	106	44	15
NKY3233	*msh5 zip1*	23	**8**	5	**19**	11	**57**	100	24	n.d.
		33	**8**	4	**9**	5	**74**	93	17	n.d.

* significantly different distributions (P<0.017) of viable spore classes (G-test) and proportion of four-viable spores (t-test) at 33°C compared to 23°C.

To verify that the improved spore viability at 33°C was due to improved chromosome segregation, we assessed the segregation of chromosome *III* in tetrads. To this end, we generated diploid strains where LacO (Y55, BR) or TetO (SK1) repeats had been inserted near the centromere of chromosome *III*. These strains also express the recombinant LacI-GFP or TetR-GFP that recognize the LacO and TetO repeats, respectively. Upon the completion of meiosis, each of the four spores in the ascus should contain a single GFP focus, provided meiosis I segregation occurred normally. In contrast, a meiosis I non-disjunction event will generate an ascus where two of the spores contain two GFP signals and two spores lack GFP altogether. In all of the three strains, meiosis I non-disjunction frequencies were decreased ∼3-fold at 33°C compared to 23°C (Figure 1C). Thus, improved chromosome segregation is the most likely explanation for the enhanced spore viability in the *msh4* and *msh5* strains at 33°C.

### Crossover frequencies are not increased on chromosome *III*

*msh4* mutants are defective in crossing over [12], [16], [26]. Therefore, we considered whether crossover formation was different at 33°C in the *msh4* mutant. Classical genetic analysis of crossing over on chromosome *III* (Figure 1D) in the four-viable spored tetrads revealed similar crossover levels in the *msh4* mutant at 23°C and 33°C (Table 3). These observations suggest that total levels of crossovers within the population of viable spores are unaffected by temperature in the *msh4* mutant.

**Table 3:** Genetic map distances in wild-type and *msh4* strains.

Strain	Temp.	Interval^a^
			*iNAT-*	*iHPH*			*iHPH-*	*LEU2*			*LEU2*	*-MAT*	
		PD	NPD	TT	cM	PD	NPD	TT	cM	PD	NPD	TT	cM
Wild type (ERY103)	23	1347	7	336	**11.2**	1435	7	245	**8.5**	828	66	799	**35.3**
	33	187	1	28	**7.9**	168	0	45	**10.6**	124	4	87	**25.8**
*msh4* (ERY137)	23	384	0	48	**5.6** *	419	1	14	**2.3** *	344	3	80	**12** *
	33	420	0	25	**2.8** *	421	0	24	**2.7** *	377	2	70	**9.1** *
	37	361	0	16	**2.1** *	366	0	17	**2.2** *	316	1	70	**9.8** *
	39	229	0	8	**1.7** *	228	0	13	**2.7** *	185	1	54	**13** *

aMap distances of genetic intervals were calculated according to Perkins, where PD is the number of four-viable spored tetrads with parental ditype, NPD non-parental ditype, and TT tetratype. cM- centiMorgans.

* The distribution of tetrad classes was significantly different from wild type (P<0.017, G-test), at the respective temperature.

### A non-exchange homeologous chromosome *III* pair displays improved chromosome segregation at 33°C compared with 23°C in the absence of *MSH4*

To determine unambiguously that *msh4* mutants have improved chromosome segregation independently of enhanced crossover frequencies, we assessed the segregation of a homeologous chromosome pair. In this diploid strain of Y55, one of the chromosome *III*s had been replaced by the co-linear chromosome from the sibling species, *S. paradoxus*. The resulting sequence divergence (10–20%) [27], [28] suppresses crossing over in ∼90% of wild-type meioses [24]. We failed to observe a single crossover in the *LEU2-MAT* interval in the inspected tetrads at 23°C or 33°C in the *msh4* strain (data not shown), suggesting that crossing over is not increased. The homeologous chromosome pair missegregates in 7.3% and 1.7% of meioses at 23°C and at 33°C respectively (Table 4). These observations support the hypothesis that temperature regulates segregation independently of modulating crossover levels on chromosome *III*.

**Table 4:** Gamete viability of strains carrying a homeologous chromosome pair.

Strain nr.	Genotype	Temp.	Category					n	% viability	% sporulation	Non-maters^a^	% NDJ^b^
		(°C)	4∶0	3∶1	2∶2	1∶3	0∶4					
ERY410	Homeologous	23	**76**	13	**11**	0	**0**	110	91	n.d.	9	8.2
	wild type	33	**65**	17	**16**	2	**0**	218	86	n.d.	15	6.9
ERY313*	Homeologous	23	**13**	4	**25**	10	**48**	96	31	n.d.	7	7.3
	*msh4*	33	**32**	36	**22**	10	**0**	59	72	n.d.	1	1.7*

* Significantly different distributions (P<0.017, G-test) at 33°C compared to 23°C.

n.d.- not determined.

anumber of two-viable spored tetrads where both spore colonies were non-mating due to containing the homeologous chromosome *III* pair.

bNon-disjunction of the homeologous chromosome *III* calculated as the number of non-maters divided by total tetrads analysed (n).

It is unclear whether temperature improves the segregation of the homeologous chromosome pairs in the presence of *MSH4*, since the homeolog pair did not display any detectable temperature-dependent decrease in its non-disjunction frequency at 33°C. In the wild-type strain, the homeolog pair missegregated in 8.2% of meioses at 23°C, similar to previous observations [24], and 6.9% at 33°C (Table 4). These observations raise the possibility that the temperature-dependent modulation of chromosome segregation might be specific to *msh4* mutants.

### A defect in meiotic progression does not select for improved viability amongst successfully sporulated cells

Progression to meiosis I in zmm mutants in the SK1 background is more severely abrogated at 33°C than at 23°C. This is accompanied by a more severe defect in crossing over at the *HIS4LEU2* hotspot at 33°C (15% of wild type) compared to 23°C (40–50% of wild type) [16]. Nevertheless, both the *msh4* and *msh5* mutants in SK1 displayed relatively good viability, particularly at 33°C (Table 2). It is therefore possible that preferential progression of the meiotic cells with relatively high accuracy of chromosome segregation may be selected. Although this could explain the phenotype in SK1, in Y55 and BR, sporulation frequencies were similar at the two temperature**s** in the msh4 strains (>45%, Table 1 and data not shown). Importantly, further decreasing the sporulation frequency of *msh4* in Y55 by increasing temperature to 37 or 39°C, does not influence viability or crossover frequencies (Tables 1 and 3).

### Deletion of *ZIP1* reduces spore viability of the *msh4* mutant at 33°C to levels observed at 23°C

Homolog pairing was also not affected by temperature. Using fluorescent *in situ* hybridization (FISH) with probes against chromosome *III* as well as *V* in the *msh4* mutant (EY137), we observed homolog pairing frequencies that were similarly high at both 23 and 33°C (>95%, data not shown), consistent with previous observations that deletion of *MSH4* or *MSH5* does not influence the juxtapositioning required for recombination in the Spo11-independent Cre/Lox system [29]. Nevertheless, homolog recognition mediated by meiotic recombination is presumably still required for the improved segregation in *msh4* since *spo11 msh4* double mutants displayed <2% spore viability (irrespective of temperature), similar to a *spo11* mutant (Table 5).

**Table 5:** Genetic requirements for spore viability of *msh4*.

Strain nr.^a^	Genotype	Temp.	Category					n	% live	% sporulation
		(°C)	4∶0	3∶1	2∶2	1∶3	0∶4			
ERY137*	*msh4*	23	**32**	8	**25**	8	**27**	1475	53	45
		33	**63**	11	**15**	3	**8**	421	79	76
ERY222	*msh4 spo11*	23	**0**	0	**1**	0	**99**	177	0.3	n.d.
		33	**0**	0	**3**	0	**97**	88	1.7	n.d.
ERY340	*zip1*	23	**24**	17	**42**	13	**4**	92	61	48
		33	**6**	18	**44**	19	**13**	90	46	32
ERY357	*msh4 zip1*	23	**31**	15	**41**	6	**7**	172	54	36
		33	**35**	9	**41**	5	**10**	108	64	24

aData for ERY137 and ERY340 from Table 4.

* Significantly different distributions (P<0.017, G-test) of viable spore classes and proportion of four-viable spores at 33°C compared to 23°C.

n.d.- not determined.

Finally, since other *zmm* mutants did not display the improvement in chromosome segregation at 33°C, we carried out epistasis analysis. *ZIP1*, which encodes the transverse element of the synaptonemal complex, is required for the temperature-mediated improvement in chromosome segregation (Table 5). The *msh4 zip1* mutant displayed similar spore viabilities at 23 and 33°C.

## Discussion

The *zmm* mutants have similar defects in crossover frequencies, however, *msh4* and *msh5* display improved chromosome segregation at 33°C. At least two observations suggest that temperature modulates chromosome segregation independently of increasing crossover frequencies. First, crossover frequencies on chromosome *III* were similar at 23 and 33°C (Table 3). Second, missegregation of the non-exchange homeolog chromosome pair was also improved at 33°C in *msh4* cells.

Although crossover frequencies are important, another consideration is the crossover position relative to the centromere, since centromere distal crossovers are less likely to facilitate biorientation of homologs, at least in *mad2* mutants of budding yeast [30]. In our dataset, crossovers falling within the *LEU2-MAT* interval that spans *CEN3* (−20 kb to +100 kb) were similar at 33°C and 23°C (Table 3), suggesting that crossovers were not being redistributed to the centromeric region at 33°C thereby promoting segregation.

The enhanced segregation at 33°C depends upon Spo11, since deleting *SPO11* in the *msh4* strains caused spore viability to mimic those observed in a *spo11* strain (Table 5). Therefore, some aspect of recombination is presumably required for chromosome segregation in the *msh4* strain.

Moreover, another ZMM protein, Zip1, is important for the temperature-mediated chromosome segregation phenotype. Although a previous study failed to observe an effect of deleting *ZIP1* affecting the segregation of a non-exchange homeologous chromosome pair [31], Zip1 might be important for chromosome segregation of homologous chromosomes that fail to crossover. Mechanisms that aid the segregation of non-exchange chromosome pairs (also originally referred to as distributive segregation) in *S. cerevisiae* are well-documented [21], [30], [32]. One hypothesis would be that homolog recognition, which depends upon Spo11-dependent recombination (noncrossover or even crossover), is followed by a Zip1-mediated segregation mechanism that occurs independent of its function in crossing over. Another possibility is that different recombination intermediates- that might support chromosome segregation- could be formed in the *msh4* mutant compared to the *zip1* mutant. Differences in the recombination phenotypes of *zmm* mutants have been reported [14], [16].

Finally, we note that at 23°C, the *zmm* mutants, including *msh4* and *msh5*, display similar reductions in spore viability. Thus, whichever mechanism(s) promotes chromosome segregation in the *msh4* strain, it requires and responds to elevated temperature (33°C).

## Materials and Methods

### Yeast genetics and sporulation conditions

All strains are listed in Supplementary Table S1 and were verified by PCR and/or Southern blot as well as meiotic segregation. Initial screening of gamete viability in *zmm* mutants was carried out in SK1 on 2% KAC-COM-agar 2% KAC, 0.22% yeast extract, 0.05% D-glucose, 0.087% complete drop-out mixture (Abdullah and Borts 2001), 2% agar, pH 7.0. This medium was also used for Y55. For BR, sporulation on solid medium was carried out on 2% KAC, 0.2% yeast extract, 0.1% D-glucose, 0.1% complete amino acid mix (Bio101), and 2% agar, pH 7.0. Spore viability was determined by standard tetrad dissection.

### Microscopy

Meiotic spreads and FISH analysis was carried out, as described [33]. GFP was visualised using a Deltavision IX70 system (Applied Precision) using the *softWoRx* software, and an Olympus Plan Apo 100×1.4 numerical aperture objective lens.

### Statistics

Map distances were calculated according to Perkins [34]. We used the Fisher exact test, t-test for proportions, or G-test for homogeneity, adjusting the P-values according to Dunn-Sidak or Tukey-Kramer methods when multiple comparisions where made.

## Supporting Information

Table S1 a iNAT and iHPH indicate the insertion of NATMX4 and HPHMX4 cassettes (GOLDSTEIN and MCCUSKER 1999) as illustrated in Figure 1D and described in HOFFMANN et al. 2005. All Y55 strains are pure Y55 constructed by transformation or crossing as described in HOFFMANN et al. 2005 and references therein.
(0.06 MB PDF)
