# Supplementary material for: Temperature-Dependent Modulation of Chromosome Segregation in msh4 Mutants of Budding Yeast
Source: PLoS One. 2009 Oct 9;4(10):e7284. doi: 10.1371/journal.pone.0007284 (PMC2757900; doi:10.1371/journal.pone.0007284)
Supplement: Table S1 — a iNAT and iHPH indicate the insertion of NATMX4 and HPHMX4 cassettes (GOLDSTEIN and MCCUSKER 1999) as illustrated in Figure 1D and described in HOFFMANN et al. 2005. All Y55 strains are pure Y55 constructed by transformation or crossing as described in HOFFMANN et al. 2005 and references therein. (0.06 MB PDF) [file pone.0007284.s001.pdf]

Supplementary Table S1. Yeast strains used in this study.

Strain	Genotype
Y97[a] (Y55) (HOFFMANN *et al.* 2005)	*MATα iNAT his4-ATC iHPH LEU2 trp5-1 cyh2-R ade1-1 lys2-c ura3-1 hoΔ::Pst*
Y128 (Y55) (HOFFMANN *et al.* 2005)	*MAT***a** *BIK1-ΔPvuII HIS4-ΔHhaI leu2-r met13-2 lys2-d ura3-1 hoΔ::Pst*
ERY103 (Y55)	*MATα iNAT his4-ATC iHPH LEU2 trp5-1 cyh2-R MET13*<br>*MAT***a** *HIS4-HhaI leu2-r TRP5 CYH2 met13-2*<br><br>*ade1-1 lys2-c ura3-1 hoΔ::Pst*<br>*ADE1 lys2-d ura3-1 hoΔ::Pst*
ERY137 (Y55)	*MATα iNAT his4-ATC iHPH LEU2 trp5-1 cyh2-R MET13*<br>*MAT***a** *HIS4-HhaI leu2-r TRP5 CYH2 met13-2*<br><br>*ade1-1 lys2-c ura3-1 hoΔ::Pst msh4Δ::KANMX4*<br>*ADE1 lys2-d ura3-1 hoΔ::Pst msh4Δ::KANMX4*<br><br>Isogenic to ERY103
Y55-2689	*MATa leu2 ade2-1 hoΔ::Pst ura3::N canR zip2::KANXM4*
Y55-2691	*MATα his4-R1 met14::URA3 cyh2-R lys2-c ade2-1 hoΔ::Pst ura3::N zip2::KANMX4*
ERY254 (Y55)	Y55-2689 × Y55-2691
ERY319 (Y55)	*MATα iNAT his4-ATC iHPH LEU2 trp5-1 cyh2-R MET13*<br>*MAT***a** *HIS4-HhaI leu2-r TRP5 CYH2 met13-2*<br><br>*ade1-1 lys2-c ura3-1 hoΔ::Pst mer3Δ::KANMX4*<br>*ADE1 lys2-d ura3-1 hoΔ::Pst mer3Δ::KANMX4*<br><br>Isogenic to ERY103
ERY320 (Y55)	*MATα iNAT his4-ATC iHPH LEU2 trp5-1 cyh2-R MET13*<br>*MAT***a** *HIS4-HhaI leu2-r TRP5 CYH2 met13-2*

	ade1-1 lys2-c ura3-1 hoΔ::Pst msh5Δ::KANMX4<br>*ADE1 lys2-d ura3-1 hoΔ::Pst msh5Δ::KANMX4*<br><br>Isogenic to ERY103
ERY340 (Y55)	*MATα iNAT his4-ATC iHPH LEU2 trp5-1 cyh2-R MET13*<br>*MAT***a** *HIS4-HhaI leu2-r TRP5 CYH2 met13-2*<br><br>*ade1-1 lys2-c ura3-1 hoΔ::Pst zip1Δ::KANMX4*<br>*ADE1 lys2-d ura3-1 hoΔ::Pst zip1Δ::KANMX4*<br><br>Isogenic to ERY103
ERY357 (Y55)	*MATα iNAT his4-ATC iHPH LEU2 trp5-1 cyh2-R MET13*<br>*MAT***a** *HIS4-HhaI leu2-r TRP5 CYH2 met13-2*<br><br>*ade1-1 lys2-c ura3-1 hoΔ::Pst*<br>*ADE1 lys2-d ura3-1 hoΔ::Pst*<br><br>*msh4Δ::KANMX4 zip1Δ::KANMX4*<br>*msh4Δ::KANMX4 zip1Δ::KANMX4*<br><br>Isogenic to ERY103
ERY423 (Y55)	*MATα iNAT his4-ATC iHPH LEU2 trp5-1 cyh2-R MET13*<br>*MAT***a** *HIS4-HhaI leu2-r TRP5 CYH2 met13-2*<br><br>*ade1-1 lys2-c ura3-1 hoΔ::Pst*<br>*ADE1 lys2-d ura3-1 hoΔ::Pst*<br><br>*msh4Δ::KANMX4 msh5Δ::KANMX4*<br>*msh4Δ::KANMX4 msh5Δ::KANMX4*<br><br>Isogenic to ERY103
ERY321 (Y55)	*MATα iNAT his4-ATC iHPH LEU2 trp5-1 cyh2-R MET13*<br>*MAT***a** *HIS4-HhaI leu2-r TRP5 CYH2 met13-2*<br><br>*ade1-1 lys2-c ura3-1 hoΔ::Pst*<br>*ADE1 lys2-d ura3-1 hoΔ::Pst*<br><br>*msh4Δ::KANMX4 spo11Δ::KANMX4*<br>*msh4Δ::KANMX4 spo11Δ::KANMX4*<br><br>Isogenic to ERY103

Y1354 (Y55)	*MAT***a** *BIK1-ΔPvuII HIS4-ΔHhaI leu2-r met13-2 lys2-d ura3-1*<br>*hoΔ::Pst CEN3::LacO-LEU2 ura3::LacI-GFP-URA3*<br>*msh4Δ::KANMX4*
Y1355 (Y55)	*MATα BIK1-ΔPvuII HIS4-ΔHhaI leu2-r met13-2 lys2-d ura3-1*<br>*hoΔ::Pst CEN3::LacO-LEU2 ura3::LacI-GFP-URA3*<br>*msh4Δ::KANMX4*
Y1356 (Y55)	*MAT***a** *BIK1-ΔPvuII HIS4-ΔHhaI leu2-r met13-2 lys2-d*<br>*MAT α BIK1-ΔPvuII HIS4-ΔHhaI leu2-r met13-2 lys2-d*<br>*hoΔ::Pst CEN3::LacO-LEU2 ura3-1::LacI-GFP-URA3*<br>*hoΔ::Pst CEN3::LacO-LEU2 ura3-1::LacI-GFP-URA3*<br>*msh4Δ::KANMX4*<br>*msh4Δ::KANMX4* (parents Y1354 and Y1355)
Y636 (BR)<br>(TSUBOUCHI and ROEDER 2005)	*MAT***a** *leu2 CEN3::LacO-LEU2 ura3::LacI-GFP-URA3*<br>*MATα leu2 CEN3::LacO-LEU2 ura3::LacI-GFP-URA3*<br>*CTF19-13MYC-KANMX4 ade2 hoΔ*<br>*CTF19-13MYC-KANMX4 ade2 hoΔ*<br>Isogenic to BR1919-8B.
Y644 (BR)	*MAT***a** *leu2 CEN3::LacO-LEU2 ura3::LacI-GFP-URA3*<br>*MATα leu2 CEN3::LacO-LEU2 ura3::LacI-GFP-URA3*<br>*CTF19-13MYC-KANMX4 ade2 hoΔ msh4::ADE2*<br>*CTF19-13MYC-KANMX4 ade2 hoΔ msh4::ADE2*<br>Isogenic to BR1919-8B.
NKY strains (SK1)<br>(BORNER *et al.* 2004)	Described in (BORNER *et al.* 2004). All isogenic to the wild-type strain.
Y660 (SK1)<br>(MARSTON *et al.* 2004)	a.k.a. A4655. *his3 ::hisG leu2 ::hisG TetO-LEU2 trp1 ::hisG pSTE5::URA3-TRP1 TetR-GFP-URA3 lys2 ho::LYS2 MAT***a**
Y821 (SK1)	*his3 ::hisG leu2 ::hisG TetO-LEU2 trp1::hisG pSTE5::URA3-*

	TRP1 TetR-GFP-URA3 lys2 ho::LYS2<br><br>*MAT***a** *msh4Δ::KANMX4*
Y1137 (SK1)	*his3 ::hisG leu2 ::hisG TetO-LEU2 trp1 ::hisG pSTE5::URA3-*<br><br>*TRP1 TetR-GFP-URA3 lys2 ho::LYS2*<br><br>*MAT*α *msh4Δ::KANMX4*
Y1259 (SK1)	<u>*his3 ::hisG leu2 ::hisG TetO-LEU2 trp1 ::hisG*</u><br>*his3 ::hisG leu2 ::hisG TetO-LEU2 trp1 ::hisG*<br><br><u>*pSTE5::URA3-TRP1 TetR-GFP-URA3 lys2 ho::LYS2*</u><br>*pSTE5::URA3-TRP1 TetR-GFP-URA3 lys2 ho::LYS2*<br><br><u>*MAT***a** *msh4Δ::KANMX4*</u><br>*MAT*α *msh4Δ::KANMX4* (parents Y821 and Y1137)
ERY 410 (Y55)	<u>*MAT*α HIS4 LEU2 S. paradoxus chr. III</u><br>*MAT***a** *BIK1-ΔPvuII HIS4-ΔHhaI leu2-r S. cerevisiae chr. III*<br><br><u>*ade1-*</u>*1* <u>*cyhR met13-*</u>*4* <u>*kar1-13*</u> <u>*ura3-N*</u> <u>*LYS2*</u> <u>*hoΔ*</u><br>*met13-2 ura3-1 lys2-d hoΔ::Pst*
ERY 313 (Y55)	<u>*MAT*α HIS4 LEU2 S. paradoxus chr. III</u><br>*MAT***a** *BIK1-ΔPvuII HIS4-ΔHhaI leu2-r S. cerevisiae chr. III*<br><br><u>*ade1-*</u>*1* <u>*cyhR met13-*</u>*4* <u>*kar1-13*</u> <u>*ura3-N*</u> <u>*LYS2*</u> <u>*hoΔ*</u><br>*met13-2 ura3-1 lys2-d hoΔ::Pst*<br><br><u>*msh4Δ::KANMX4*</u><br>*msh4Δ::KANMX4*

Supplementary References.

BORNER, G. V., N. KLECKNER and N. HUNTER, 2004 Crossover/noncrossover differentiation, synaptonemal complex formation, and regulatory surveillance at the leptotene/zygotene transition of meiosis. Cell 117: 29-45.

GOLDSTEIN, A. L., and J. H. MCCUSKER, 1999 Three new dominant drug resistance cassettes for gene disruption in Saccharomyces cerevisiae. Yeast 15: 1541-1553.

HOFFMANN, E. R., E. ERIKSSON, B. J. HERBERT and R. H. BORTS, 2005 *MLH1* and *MSH2* promote the symmetry of double-strand break repair events at the *HIS4* hotspot in *Saccharomyces cerevisiae*. Genetics 169: 1291-1303.

MARSTON, A. L., W. H. THAM, H. SHAH and A. AMON, 2004 A genome-wide screen identifies genes required for centromeric cohesion. Science 303: 1367-1370.

TSUBOUCHI, T., and G. S. ROEDER, 2005 A synaptonemal complex protein promotes homology-independent centromere coupling. Science 308: 870-873.
